# Early and Late De Novo Tumors after Liver Transplantation in Adults: The Late Onset of Bladder Tumors in Men

**DOI:** 10.1371/journal.pone.0065238

**Published:** 2013-06-13

**Authors:** Umberto Maggi, Dario Consonni, Matteo Angelo Manini, Stefano Gatti, Francesco Cuccaro, Francesca Donato, Grazia Conte, Pier Alberto Bertazzi, Giorgio Rossi

**Affiliations:** 1 UO (Unità Operativa) Chirurgia Generale e Trapianti di Fegato–Fondazione IRCCS (Istituto di Ricovero e Cura a Carattere Scientifico) Ca’ Granda Ospedale Maggiore Policlinico di Milano, Milano, Italy; 2 Department of Digestive and Hepatobiliary Surgery, AP-HP (Assistance Publique-Hôpitaux de Paris), UFR (Unité de formation et de recherche) de médecine de l'Université Paris, Paris, France; 3 UO Chirurgia Generale e Trapianti di Fegato–Fondazione IRCCS Ca’ Granda Ospedale Maggiore Policlinico di Milano, Milano, Italy; 4 UO Epidemiologia–Fondazione IRCCS Ca’ Granda Ospedale Maggiore Policlinico di Milano, Milano, Italy; 5 Azienda Sanitaria Locale BAT (Barletta, Andria, Trani), Unità Operativa Epidemiologia e Statistica Barletta, Barletta, Italy; 6 Gastroenterology, Fondazione IRCCS Ca' Granda Ospedale Maggiore Policlinico, University of Milan, Italy; 7 Department of Clinical Sciences and Community Health, Università degli Studi di Milano, Milan, Italy; 8 General Surgery and Liver Transplantation Unit, Department of Surgical Sciences, University of Milan, Milan, Italy; University of Navarra School of Medicine and Center for Applied Medical Research (CIMA), Spain

## Abstract

**Background:**

De novo tumors (DNT) after liver transplantation (LT) represent a growing concern.

**Patients and Methods:**

We analyzed the incidence of DNT, type, time of onset, risk factors and mortality (as of 2010) in 494 adult patients transplanted in the last 26 years (1983–2009).

**Results:**

DNT occurred in 41 (8.3%) of the patients. The Standardized Incidence Ratio (SIR) compared with the Italian population was 1.8. There was a higher incidence in males (SIR 2.0), an expected extremely high rate of Kaposi’s sarcoma (SIR 127.95) and unexpected higher rates of tumors of the bladder in males (SIR 3.3). The incidence of DNT was higher within the first two years of LT (SIR 2.7) for Kaposi’s sarcoma (SIR 393.3) and after 10 years (SIR 1.7) for bladder tumors (SIR 10.6). Multivariate analysis identified alcoholic cirrhosis (HR = 3.0, 95% CI = 1.2–7.8) and sclerosing cholangitis (HR = 3.5, 95% CI = 1.1–11.3) in the recipient as main risk factors for the occurrence of DNT.

**Conclusions:**

Surveillance protocols for DNT must be specifically oriented to patients transplanted for alcoholic cirrhosis and sclerosing cholangitis. They should focus on early detection of Kaposi’s sarcomas, and more remarkably, on late development bladder tumors in men after LT.

## Introduction

Liver transplantation (LT) is presently the gold standard therapy for patients with end-stage liver disease. In recent years, the results of this procedure have shown a significant and steady improvement in long term survival [1]. Due to these improving results, early and late problems still occur after liver transplantation [1]. Among late complications, recurrences of primary diseases may be predictable. De Novo Tumors (DNT) can arise at different times [2], may be more unpredictable and therefore need specific attention. Their rate and time of onset as well as pre-disposing etiological factors should be identified to help screening and treatment. Published data on DNT arising after liver transplantation indicate a cumulative incidence of 5–6% at 5 years, 13–20% at 10 and 16% at 20 years [3], [4].

The main aim of this study was to compare the rates of different DNTs among patients who have had a liver transplant with those of the general population and to evaluate the DNT risk based on time from transplantation.

## Materials and Methods

### a) Patients

The Ethics Committee of our institution –Fondazione IRCCS Ca’ Granda Ospedale Maggiore Policlinico of Milan - was notified of this study. According to Italian law (Gazzetta Ufficiale, serie generale, n.76, p. 67–74) no specific approval is needed for retrospective studies.

Informed consent was obtained as usual for medical, surgical, radiological treatments, not specifically for this retrospective study. Patients gave written consent for every procedure performed in the hospital including treatment of data for medical purposes.

Data from 571 adult patients with 644 liver grafts spanning a period from 1983 to 2009 were analysed. The detection of some tumors occurred as early as the second month after LT and thus we excluded 57 patients (with 67 LTs) with a post-operative follow-up time ≤30 days.

Patients transplanted for primary hepatic tumors (i.e. hepatomas- HCC) were included, assuming that these tumors did not influence the occurrence of DNTs.

Cyclosporine was the main immunosuppressive drug from 1983 through march 1993. Thereafter it was often replaced by Tacrolimus except for HCV patients. OKT3 was sometimes used. The policy of our Center was to treat HCV patients with Cyclosporine.

No specific protocol for detecting de novo tumors was employed. In the post-operative period patients underwent physical, radiological (in the form of an abdominal ultrasound) and biochemical assessment. In suspected cases of DNT measurement of tumor markers (alpha-1-fetoprotein, Ca 19–9, Carcinoembryonic antigen) were included as part of the biochemical investigation. All data was entered into a retrospective database specifically for this study. Cancer incidence and mortality follow-up was performed by collecting clinical information during periodic pre-scheduled visits from patients/relatives or by periodic active phone/fax contact.

The follow-up ended on April 30, 2010. Twenty patients were lost to follow-up bringing the final cohort of patients included in the study to 557 grafts in 494 adult patients: 437 patients were transplanted once, 51 twice, and 6 three times.

New primary cancer diagnoses were coded according to the International Classification of Diseases and Related Health Problems, 10th revision (ICD-10). Non-melanoma skin cancers were excluded from analysis in the study. The recurrence of HCC was not considered an event in our study. For patients residing in Milan, completeness of cancer identification was checked by record-linkage with official hospital admissions databases in the period 2000–2008 and with the Milan cancer registry.

### b) Statistical Analysis

The overall and tumor-specific cancer incidence in the cohort of transplanted patients was compared with the expected incidence according to national cancer incidence rates (provided by the Italian Association of Cancer Registries, AIRTum, http://www.registri-tumori.it/cms/) specific for gender, calendar period, and age. Person-years at risk for cancer incidence were computed from 30 days after the date of transplant to the date of cancer diagnosis, date of death, date of last follow-up visit or to the date of termination of the study (April, 30, 2010), whichever came first. For each cancer site, we calculated Standardized Incidence Ratios (SIR) as the ratio of observed (Obs) to expected (Exp) number of cancer cases; 95% CI of SIRs were calculated using the exact method based on the Poisson distribution [5]. SIR patterns by gender and time since LT were evaluated.

The cumulative DNT risk was analyzed using the Kaplan-Meier estimator [6]. We estimated the association between DNT risk and selected donor, transplant, and recipient characteristics by calculating Hazard Ratios (HR) and their 95% confidence intervals (95% CI) with univariate Cox proportional hazard models [6]. The covariates of the priori interests and those emerging from univariate analyses with statistical significance were then included in a multiple regression Cox model to calculate adjusted HRs. Several demographic, clinical and biochemical data related to donor, transplant and recipients were collected and analysed.

Mortality of DNT patients was compared with that of the not DNT patients using Kaplan-Meier estimator and Cox regression. Person-years from 30 days since LT until DNT diagnosis were considered as cancer-free person-years [7].

Finally, we compared all mortality causes in transplanted patients with those in the general Italian population by calculating Standardized Mortality Ratios (SMR) using mortality rates specific for calendar period (5 year categories), gender, and age (5 year classes). Statistical analyses were performed using Stata 11 (StataCorp LP, Texas, USA) [8].

## Results

The average follow-up time in the 494 pts was 7.2 years (standard deviation 5.6 years), (range from 33 days to 22 years). Overall, the person-years at risk from LT to de-novo occurrence or censoring were 3,761 Years.

Recipient characteristics are reported in Table 1.

**Table 1:** Clinical characteristics of 494 adult recipients who underwent primary liver transplantation, 1983–2009.

Demographic and clinical characteristics of Recipients	494 patients	
	n	%
**Sex**		
Male	334	58.3
Female	160	27.9
**Age (mean, SD)**	46.8, 10.8	
median (range**)**	49.0 (19–66)	
**Geographic area of birth**		
Northern Italy	197	43.8
Central Italy	16	3.6
Southern and insular Italy	215	47.8
Out of Italy	22	4.9
Unknown	44	
**Indication to transplantation**		
Virus related cirrhosis	201	40.7
Hepatic Tumors (HCC+CCC)	108	21.9
Alcoholic cirrhosis	36	7.5
Sclerosing cholangitis	20	3.8
Primary Biliary cirrhosis	35	7.3
Fulminant/subfulminant hepatic failure	27	5.5
Others	67	13.4
**Virus induced disease**	314	63.6
HCV (Hepatitis C virus)	181	36.6
HBV (Hepatitis B virus)	151	30.6
**Starting immunosuppression**		
Cyclosporine	281	56.9
Tacrolimus	155	31.4
Not available	58	11.7
**Retransplantations**	57	11.5
**Causes of death**	N = 131	22.9
Sepsis	30	22.9
Miscellaneous	28	21.4
Non tumor recurrence	20	15.3
Tumor recurrence	15	11.5
De novo tumors	**41**	**10.7**
Technical failures	12	9.2
Primary o Delayed non function	5	3.8
Rejection	4	3.1
Perioperative death. after long follow-up	3	2.3
**Follow-up time** (years), mean, SD	7.2, 5.6	
median (range)	5.6 (33 days - 22 years)	

### Incidence of de Novo Tumors

In 494 liver transplant recipients, 50 DNTs (10.1%) were found, including 9 (1.8%) non-melanoma skin cancers(Table 2). Excluding non-melanoma skin cancers, there were 41 (8.3%) patients with DNTs (31 men and 10 women) with average age at time at diagnosis of 55 years (standard deviation 9). When compared with the general Italian population, cancer incidence was higher (SIR 2.00) in males and slightly increased (SIR 1.31) in females. Both genders had extremely elevated SIRs for Kaposi’s sarcoma (7 cases overall against only 0.05 expected- per100,000). Male gender showed elevated SIRs for several sites, including upper aerodigestive, breast, bladder and hematologic neoplasms (notably lymphomas). In females, elevated SIRs (based on only one case) for upper aerodigestive, stomach,uterus and hematologic neoplasms, and (2 cases) for lung cancer.(SIR 5.3).

**Table 2:** Observed (Obs) and expected (Exp) cases of de-novo tumors, Standardized Incidence Ratios (SIR), and 95% Confidence Intervals (95% CI) among 494 adult recipients who underwent primary liver transplantation, 1983–2009, by gender. Reference: pool of Italian cancer registries.

	Males				Females				Total			
Cancer site/type (ICD-10 code)	Obs	Exp	SIR	95% CI	Obs	Exp	SIR	95% CI	Obs	Exp	SIR	95% CI
All cancers but skin (C00–C43, C44–C 97)	31	15.4	2.0	1.4–2.9	10	7.6	1.3	0.6–2.4	41	23.0	1.8	1.2–2.4
Upper aerodigestive (C00–C14, C30–C33)	3	1.0	2.9	0.6–8.4	1	0.1	8.7	0.2–48.7	4	1.2	3.4	0.9–8.8
Stomach (C16)	0	0.7	0		1	0.2	5.3	0.1–29.8	1	0.9	1.2	0.03–6.5
Colon (C18)	3	1.3	2.2	0.5–6.5	0	0.6	0		3	1.9	1.6	0.3–4.6
Lung (C33–C34)	4	2.5	1.6	0.4–4.2	2	0.4	5.3	0.6–19.0	6	2.8	2.1	0.8–4.6
Kaposi’s sarcoma (C46)	6	0.05	120.0	44.0–261.0	1	0.005	212.7	5.4–1185.3	7	0.05	127.9	51.4–263.5
Breast (C50)	1	0.04	22.6	0.6–126.1	2	3.0	0.7	0.08–2.4	3	3.0	1.0	0.2–2.9
Vulva (C51.9)*					1	–			1	–		
Uterus, cervix (C53)					1	0.2	5.7	0.1–31.9	1	0.2	5.7	0.1–31.9
Prostate (C61)	4	2.5	1.6	0.4–4.1					4	2.5	1.6	0.4–4.1
Bladder (C67)	5	1.5	3.3	1.1–7.6	0	0.2	0		5	1.7	2.9	1.0–6.9
Central nervous system (C69–C72)	1	0.3	3.4	0.1–19.1	0	0.1	0		1	0.4	2.4	0.1–13.5
Hematologic cancers (C81–C96)	4	1.2	3.3	0.9–8.4	1	0.5	1.9	0.1–10.8	5	1.7	2.9	0.9–6.7
Lymphomas (C81–C85, C96)	4	0.7	5.9	1.6–15.1	0	0.3	0		4	1.0	4.1	1.1–10.4
Non–Hodgkin’s lymphoma (C82–C85, C96)	3	0.6	5.0	1.0–14.7	0	0.3	0		3	0.9	3.5	0.7–10.2
Hodgkin’s disease (C81)	1	0.1	12.5	0.3–69.4	0	0.04	0		1	0.1	8.2	0.2–45.4
Leukemia (C91–C95)	0	0.3	0		1	0.1	8.2	0.2–45.5	1	0.5	2.2	0.1–12.3

Reference: pool of Italian cancer registries.

* Reference rates not available.

The highest incidence of DNT (SIR 2.7) was found within the first two years of LT (Table 3). Five of the 11 early DNTs were KS (2 of them within 2 months of LT) with an extremely elevated SIR(393.3). Among the six remaining cancers occurring within 2 years of LT, we observed one case of stomach, lung, CNS, and NHL each, with SIRs ranging from 1.9 to 12.3 and two colon cancers with a SIR of 6.1. 2–9 years after LT, there was a six-fold excess of upper aerodigestive (laryngo-pharyngeal cancers) cancers, KS, and prostate cancer. Ten years after LT there was a clear excess of bladder cancers with five cases (2 between 10 and 15 years and 3 after 15 years from LT) (SIR 10.6), and of cervix (SIR 22.6) and hematologic cancers (SIR 4.3), but based on a few cases.

**Table 3:** Observed (Obs) and expected (Exp) cases of de-novo tumors, Standardized Incidence Ratios (SIR), and 95% Confidence Intervals (95% CI) among 494 adult recipients who underwent primary liver transplantation, 1983–2009, by years since liver transplant.

	<2 years				2–9 years				≥10 years			
Cancer site/type(ICD-10 code)	Obs	Exp	SIR	95% CI	Obs	Exp	SIR	95% CI	Obs	Exp	SIR	95% CI
All cancers but skin (C00–C43, C44–C97)	11	4.1	2.7	1.3–4.8	19	12.4	1.5	0.9–2.4	11	6.5	1.7	0.8–3.0
Upper aerodigestive (C00–C14, C30–C33)	0	0.3	–	–	4	0.6	6.2	1.7–16.1	0	0.3	–	–
Stomach (C16)	1	0.2	6.3	0.2–35.0	0	0.5	–	–	0	0.2	–	–
Colon (C18)	2	0.3	6.1	0.7–21.9	0	1.0	–	–	1	0.6	1.8	0.1–10.1
Lung (C33–C34)	1	0.5	1.9	0.05–10.6	4	1.5	2.6	0.7–6.7	1	0.8	1.2	0.03–7.0
Kaposi’s sarcoma (C46)	5	0.01	393.3	127.7–917.9	2	0.03	65.5	7.9–236.6	0	0.01	–	–
Breast (C50)	0	0.5	–	–	2	1.6	1.2	0.1–4.5	1	0.9	1.1	0.03–6.3
Vulva (C51.9)*	–	–	–	–	1	–	–	–	–	–	–	–
Uterus, cervix (C53)	0	0.03	–	–	0	0.1	–	–	1	0.05	22.2	0.5–123.8
Prostate (C61)	0	0.4	–	–	4	1.3	3.0	0.8–7.8	0	0.8	–	–
Bladder (C67)	0	0.3	–	–	0	0.9	–	–	5	0.5	10.6	3.4–24.7
Central nervous system (C69–C72)	1	0.1	12.3	0.3–68.4	0	0.2	–	–	0	0.1	–	–
Hematologic cancers (C81–C96)	1	0.3	3.0	0.1–16.7	2	0.9	2.1	0.3–7.7	2	0.5	4.3	0.5–15.7
Lymphomas (C81–C85, C96)	1	0.2	5.2	0.1–28.7	2	0.5	3.7	0.5–13.5	1	0.3	4.0	0.1–22.2
Non-Hodgkin’s lymphoma (C82–C85, C96)	1	0.2	6.0	0.2–33.7	1	0.5	2.1	0.05–12.0	1	0.2	4.4	0.1–24.7
Hodgkin’s disease (C81)	0	0.03	–	–	1	0.07	14.5	0.3–80.9	0	0.03	–	–
Leukemia (C91–C95)	0	0.1	–	–	0	0.2	–	–	1	0.1	8.3	0.2–46.0

Reference: pool of Italian cancer registries.

There was also a periodic shift in the occurrence of a predominant malignancy. From 1986–1991 there was a higher rate of colon cancer (SIR 57.8), from 1992–1996 of lung (SIR 6.22) cancer and lymphomas (SIR 13.6) and from 2006 a higher rate of Kaposi’s sarcomas (SIR 270.7) and bladder tumors (SIR 6.69).

### Specific Aspects: Bladder Cancers

Among five patients with urothelial (transitional cell) bladder cancers, two tumors were discovered after the onset of gross hematuria whereas the others during routine abdominal ultrasound exams. Three patients had early stage tumors, whereas two had T2 and T3N2 tumors, respectively. Two patients underwent a Transurethral Resection of a Bladder Tumor (TURBT), and two a radical cystectomy with extended pelvic lymph node dissection and ileal conduit urinary diversion. One patient underwent a TURBT for a T2 cancer, but then was scheduled for major surgery. Unfortunately he died before the surgical treatment.

### Risk Factors

The cumulative risk (Kaplan-Meier failure function) of DNT after LT was 8.9% (95% CI 6.2–12.7%) at 10 years and 22.8% (95% CI 15.2–33.3%) at 20 years. In the univariate Cox regression analyses only male gender, alcoholic cirrhosis, and sclerosing cholangitis were found to be predictors for development of DNT.

The covariates of priori interests and those emerging from univariate analyses (age and sex of recipient and indications for LT) were then included in a multiple Cox model to calculate adjusted HRs. Alcoholic cirrhosis (HR = 3.03, 95% CI 1.17–7.84, p = 0.02) and sclerosing cholangitis (HR = 3.51, 95% CI = 1.09–11.34, p = 0.03) were confirmed as independent risk factors.

### Mortality

**Figure 1:**
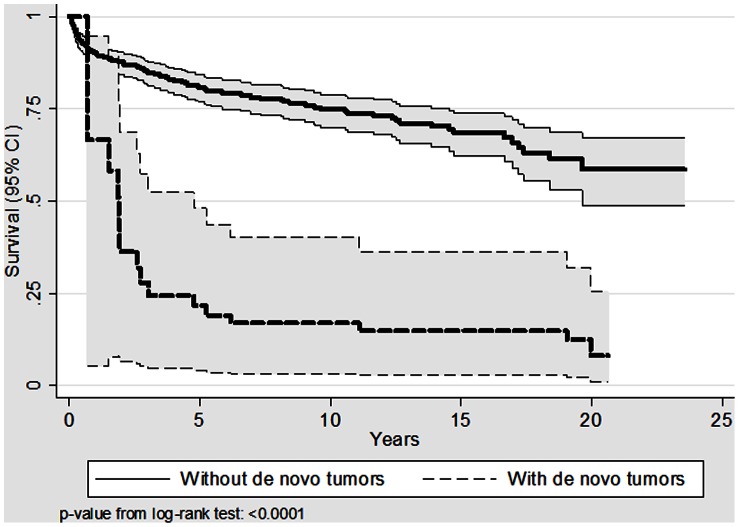
Kaplan-Meier survivor functions (thick lines) and 95% confidence intervals (95% CI, thin lines) after liver transplantation or DNT onset.

There were 14 deaths among patients with DNT and 119 among patients without DNT. Survival was significantly different (Figure 1). The gender- and age-adjusted HR of mortality in DNT patients was 4.4 (95% CI: 2.4–8.0) compared to patients without DNT. There were no differences (p = 0.71) in survival between patients with different kinds of DNT (Upper Aerodigestive, Lung tumors, Kaposi’s sarcomas and others) (data not shown).

Compared to the general Italian population, there was a dramatic reduction in mortality over time in transplanted patients both without (standardized Mortality Ratios –SMR – from 59.7 in 1983–1989 to 2,9 in 2005–2010), and with DNT (SMR from 269.2 in 1983–1989 to 5.2 in 2005–2010) compared to the Italian Mortality Rates (data not shown).

## Discussion

This is the largest and longest single centre Italian collection of DNT after LT series to date.

DNT represents a complication that has increasingly gained importance, partly due to improvements of liver transplantation [9]. The only available international register from the centers of liver transplantation, the Israel Penn International Transplant Tumor Registry (IPITTR) [10], contains valuable information on this subject but provides no data on patient follow-up and risk rate for DNT occurrence in transplanted patients [11]. Studies reported in the literature are quite heterogeneous as, there was neither standardization among the series of patients who presented with DNT nor between the analytical methods. Other differences include the number of patients, ranging from 325 to 6846 [2], [19], [10], [11], [12], [13] and duration of observation time – from 5 to 27 years - in different periods ranging form 80s to 2006 [2], [10], [11], [14], [3], [15], that could affect the results.

Distinct from most other European studies [16], [17], [18] with the exception of the series of Collett and Al. (19), the follow-up of our patients is quite long, as our first liver transplant was performed in 1983, allowing a broader perspective on this problem.

The incidence of DNT in patients undergoing liver transplantation varies between 3 and 26% [9], [12], [3] and increases with duration of follow-up. The cumulative risk of DNT has been estimated to be 6%,20% and 55% at 5, 10 and 15 years respectively [20], or 3%, 5%, 13% and 16% at 1,5,10 and 20 years respectively [3].

In the few studies that have calculated SIRs for DNT the reported figures ranges from 2.2 to 4.0 [2], [19], [11], [3], [15], [20].

The incidence of DNT in our cohort of patients was 8.3%. The SIR compared to the Italian cancer registries was 1.8. Our rates and SIR were slightly lower compared to other reported Italian series [2], [11]. This difference has no clear explanation. Relative to patients studied in the Baccarani’s paper [2] our population is younger and more frequently coming from Southern Italy.

In the published literature males appear to be more affected than females (ratio 3.29 vs 2.35) [12]. In our series, males presented an overall higher SIR for DNT than women (2 vs 1.3). Females had higher SIRs for cancers of the lung and Kaposi’s sarcoma whereas men had higher SIRs for cancers of the bladder, colon, and lymphomas.

The age of onset of cancers has been reported to be similar between transplanted patients and the general population [10] according to a recent large study of the Israeli population by Miao. Aberg et al reported that 80% of DNT after liver transplantation affects individuals between 45 and 74 years [3]. This view is supported by Haagsma [4] and Fung [21] who note that there is a positive correlation between incidence of cancer and age over 40 years. Conversely, it appears that there is a negative correlation between lymphoproliferative disorders (PTLD) and age.

In our series, the age range associated with a higher frequency of tumors was 55–59 years. This is consistent with the fact that in the general population the incidence of cancer increases with age. However, if we consider the SIR, the greatest increase if compared to the general population, is between 20 and 24 years - in relation to Kaposi's sarcoma - and between 40 and 44 years.

Viral infections appear to contribute to the development of DNT. In immunocompetent subjects, cervical cancer has been linked to Human Papilloma Virus infections, and Kaposi's sarcoma with Human Herpes Virus 8. Infection with Epstein Barr Virus is the main risk factor for developing lymphoproliferative disorders [12], [13], [18], nasopharyngeal cancer and some types of leiomyosarcoma. HCV has a controversial role [12].

All studies [2], [19], [11], [13], [22], [23], [24] agree that among primary hepatic diseases leading to liver transplantation, alcohol seem to be related to an increased rate of DNT, particularly skin cancers, cancers of the oral cavity, larynx and esophagus. Smoking has been recognized by several authors as a general [25], [26], [27] and specific risk factor for esophageal [12], skin [28] and lung [29] cancers. Unfortunately, one limitation of this study is that we were not able to evaluate smoking as a risk factor for DNT.

Delcò [30] found a significant correlation between primary sclerosing cholangitis and cancer, and specifically with colorectal cancer. In our series only one out of three colon cancers occurred in a patient transplanted because of a primary sclerosing cholangitis; however primary sclerosing cholangitis and alcoholic cirrhosis confirmed a strong statistical relationship with the onset of DNT.

With regard to immunosuppressive drugs, though there are some confirmations of the association between the use of cyclosporin and non-melanoma skin cancers [31], in our series a comparison of the two calcineurin inhibitors with the development of DNT showed no statistical difference. In addition, the use of monoclonal antibodies, such as OKT3, also failed to show any statistical significance.

We also observed an higher rate of lymphomas in the early years of transplantation (1991–1996), but of kaposi’s sarcoma and bladder tumors after 2006. No specific correlation with initial immunosuppressive therapy concerning calcineurin inhibitors was found even though treatment with cyclosporine was more frequent in the early years. The influence of other immunosuppressive drugs such as Mycofenolate, azathioprine and boluses of corticosteroids could not be studied.

In general, the published literature [3], [9], [11], [12], [32], [33], [34] agree that skin cancers, Kaposi's sarcoma (KP) and post-transplant lymphoproliferative disorders (PTLD) are the most frequent DNT. Also, in our study haematological/viral cancers showed the highest rate (29% of all DNT).

Some authors agree that the highest rate of onset of tumors is in the first year after transplantation, probably due to a greater initial immunosuppression. Our study confirms the expected early appearance of viral/lymphatic tumors. It can be postulated that early viral/lymphatic tumors may be stimulated by early immunosuppression whereas other tumors may need a longer time of stimulation by immunosuppression: so some Authors [10] report that DNT develop after a time interval ranging from 39 to 77 months post-transplant, that is within 6 years after LT, or, according to a Finnish study [3], after an average time of is 61 months (5 years). Other Authors [10] found that cancers of the colon and rectum generally arise later. One advantage of our study was the long time span. That allowed observation of very late DNTs. particularly cancers of the bladder in males which had their onset many years after transplantation. It can be speculated that the bladder in males is a late target organ of a long lasting previous immunosuppression.resulting in cancer. It is not clear why bladder cancer happens mostly in men. The late increase of bladder tumors has not been previously clearly described in LT patients and deserve attention. On the basis of this finding, new follow-up protocols should be developed. However, further confirmations and explanations are needed.

The study has some limitations. The analysis of the influence of immunosuppressive drugs had to be limited to calcineurine inhibitors and to monoclonal antibodies, only. Indeed amount of steroids, azatiophrine and mycophenolate doses were initially calculated but that study soon resulted too complicated because of dose and timing variations occurred through the years, as it often happens after LT. Such a study can be easily done for short periods, but not for long-term follow-ups.

The usual blood target levels for calcineurine inhibitors were applied (Tacrolimus levels targeted between 8–12 ng/ml within the first 90 days, then lowered; Cyclosporin levels around 200–300 ng/ml within the first 90 days). The calculation of the entire load of Cyclosporine or Tacrolimus that each patients received all along the years was attempted but this analysis is very complex. We considered OKT3, as that is a very powerful immunosuppressive drug that is expected to influence the onset of tumors as it influences the onset of septic complications. However, in our analysis no directly correlation was shown between OKT3 and DNT. This result can be explained by a poor influence of an “acute” immunosuppressive load on the onset of DNT after LT.

As already reported before, an other limitation of this study is that we were unable to evaluate smoking history in our patients. In fact, due to study design concerning data even from the ‘80s and ‘90s, some patients’ characteristics of the medical history could not be retrieved. In Italy the percentage of daily smokers is 24,2% and higher rates can be found among patients with alcoholic cirrhosis. As tobacco consumption in our patients is similar to that reported in other Western countries, and that the medical literature never mentioned specific observations on bladder tumors to date, we can argue that smoking history is probably not involved as ethiologic factor of DNT. Thus our finding of high rates of late onset bladder cancers remains unexplained.

In recent years, there has been a dramatic decrease in mortality in transplanted patients with and without DNT, probably due to an improvement in treatments of liver transplantation and cancer. However, the onset of a DNT still heavily influences the patient outcome. The analysis of post LT survival according to different DNT failed to show any statistical significance among them in this study (p = 0.71). It is the authors’ opinion that the best approach to DNT appears to be a very early detection through appropriate screening protocols. Specifically, whereas detection of Kaposi’s sarcoma and lymphomas should be the focus in the early years after LT, 10 years after LT screening should also focus on the detection of urological tumors.

### Conclusions

This is the largest and longest Italian monocentric study published so far on DNT occurrence after LT. Males were more affected. As expected, we observed increased risks of Kaposi’s sarcoma (in particular in the first 2 years after LT) and of tumors of the lymphatic system. Interestingly, we observed an increased incidence of bladder tumors in males.

Multivariate analysis of risk factors identified alcoholic cirrhosis and sclerosing cholangitis in the recipient as the main risk factors for the onset of DNT. The use of tacrolimus and cyclosporine as initial therapy showed no differences with regard to appearance of DNT.

The risk of mortality at the onset of DNT adjusted for recipient age and sex, was 4–5 times increased. No differences in survival was demonstrated among different DNT.

In conclusion, screening protocols for DNT should be specifically oriented to patients transplanted for alcoholic cirrhosis and sclerosing cholangitis. The focus should be, as expected, on Kaposi’s sarcomas early after LT, and, more remarkably, as never specifically mentioned before, on bladder tumors in men after 10–15 years from LT.
